# The EMT-induced lncRNA NR2F1-AS1 positively modulates NR2F1 expression and drives gastric cancer via miR-29a-3p/VAMP7 axis

**DOI:** 10.1038/s41419-022-04540-2

**Published:** 2022-01-26

**Authors:** Dandan Li, Mengjie Xu, Zidi Wang, Pan Huang, Congcong Huang, Zhen Chen, Gaijuan Tang, Xingji Zhu, Mengyu Cai, Shanshan Qin

**Affiliations:** 1 Hubei Key Laboratory of Embryonic Stem Cell Research, School of Basic Medical Sciences, Hubei University of Medicine, 442000 Shiyan, Hubei P.R. China; 2 Laboratory of Tumor biology, Academy of Bio-Medicine Research, Hubei University of Medicine, 442000 Shiyan, Hubei P.R. China; 3 Department of Endocrinology, Taihe Hospital, Hubei University of Medicine, 442000 Shiyan, Hubei P.R. China; 4 College of Plant Protection, Northwest A&F University, 712100 Yangling, China

**Keywords:** Gastric cancer, Prognostic markers, Long non-coding RNAs, Epithelial-mesenchymal transition

## Abstract

Deregulated lncRNAs play critical roles in tumorigenesis and tumor progression. NR2F1-AS1 is an antisense lncRNA of NR2F1. However, the biological function of NR2F1-AS1 in gastric cancer (GC) remains largely unclear. In this study, we revealed that NR2F1-AS1 and NR2F1 were both positively correlated with the degree of malignancy and predicted poor prognosis in two independent GC cohorts. Besides, NR2F1-AS1 and NR2F1 can respond to Epithelial-to-mesenchymal transition (EMT) signaling in GC, since their expression was increased by TGF-beta treatment and decreased after stable overexpression of OVOL2 in GC cell lines. NR2F1-AS1 and NR2F1 were highly co-expressed in pan-tissues and pan-cancers. Depletion of NR2F1-AS1 compromised the expression level of NR2F1 in GC cells. Furthermore, NR2F1-AS1 knockdown inhibited the proliferation, migration, invasion and G1/S transition of GC cells. More importantly, transcriptome sequencing revealed a novel ceRNA network composed of NR2F1-AS1, miR-29a-3p, and VAMP7 in GC. The overexpression of VAMP7 predicted poor prognosis in GC. Rescue assay confirmed that NR2F1-AS1 promotes GC progression through miR-29a-3p/VAMP7 axis. Our finding highlights that the aberrant expression of NR2F1-AS1 is probably due to the abnormal EMT signaling in GC. LncRNA NR2F1-AS1 plays crucial roles in GC progression by modulating miR-29a-3p/VAMP7 axis, suggesting that NR2F1-AS1 may serve as a potential therapeutic target in GC.

## Introduction

Gastric cancer (GC) is a heterogeneous tumor with the fourth highest mortality rate worldwide [1]. According to the latest global cancer burden data report released by the International Agency for Research on Cancer (IARC), there will be approximately 1.089 million new cases of gastric cancer and 769,000 deaths worldwide in 2020 [2, 3]. Due to the inconvenience of early diagnosis of GC, many patients are diagnosed at advanced stages [4]. Therefore, it is urgent and necessary to develop new biomarkers or strategies to improve the early diagnosis of GC.

Metastasis is the leading cause of cancer-related deaths, especially in stomach cancer. In 2015, Cristescu et al. have reported that GC patients with EMT molecular subtype possess worst prognosis [5]. Similarly, in 2018, Oh et al. also found that GC patients with mesenchymal phenotype possessed poorer prognosis than GC patients with intestinal phenotype [6]. Tumor metastasis are the result of a complex process that involves local invasion, intravasation, transport, extravasation, micro-metastasis formation and colonization [7]. The metastatic cascade is a multifaceted process, in which EMT mediates the initial transformation from benign to invasive carcinoma [8]. EMT, an evolutionarily conserved program of cellular plasticity, allows polarized, immotile epithelial cells to loosen their cell-cell adhesion, detach from neighboring cells, and to convert into motile mesenchymal cells [9]. The occurrence of EMT requires a variety of extracellular signals, such as transforming growth factor, fibroblast growth factor, hepatocyte growth factor, and epidermal growth factor and chemokines [10]. Once the EMT program is started, remarkable changes would be observed in the expression of the EMT-related genes [11, 12]. These EMT-related genes eventually have a profound effect on cell morphology and function [13, 14]. However, most of the existing research focuses on the coding genes caused by EMT, and the biological functions of EMT-related lncRNA are relatively rarely reported.

In this study, we identified an antisense lncRNA NR2F1-AS1 that induced by EMT in GC. NR2F1-AS1 was positively correlated with the degree of malignancy and predicted poor prognosis in two independent GC cohorts. Transcriptome sequencing revealed a novel ceRNA network composed of NR2F1-AS1, miR-29a-3p, and VAMP7 in GC. In vitro experiments confirmed that NR2F1-AS1 promotes GC metastasis through the miR-29a/VAMP7 signal axis. Therefore, targeting the NR2F1-AS1/miR-29a-3p/VAMP7 axis could be a new potential strategy for the treatment of GC.

## Results

### NR2F1-AS1 and NR2F1 are clinically correlated with poor prognosis in GC

**Fig. 1:**
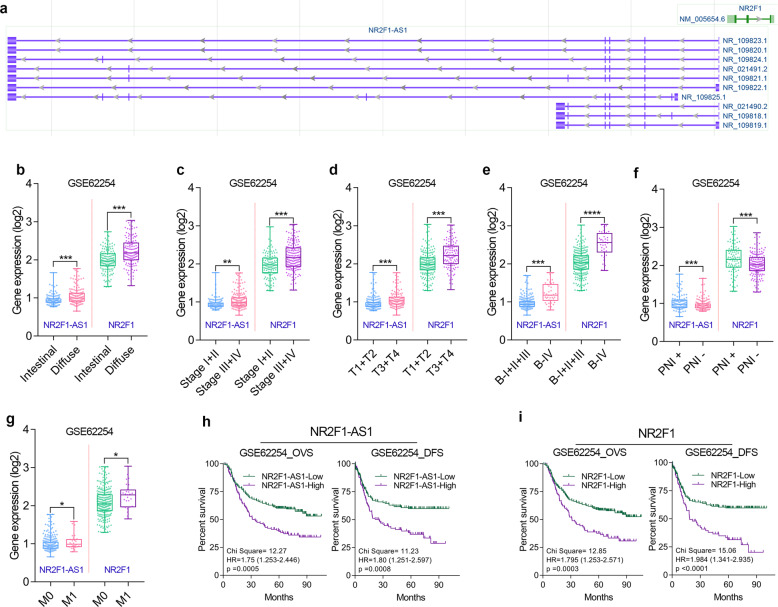
The clinical significance of NR2F1-AS1 and NR2F1 was analyzed in the GSE62254 cohort. **a** Antisense lncRNA NR2F1 has multiple types of transcripts according to the annotation of NCBI. **b** Difference in expression levels of NR2F1-AS1 and NR2F1 between intestinal and diffuse GC tissues. **c**–**e** NR2F1 and NR2F1-AS1 expression level in different Pathologic stages, T-stages and Borrmann-stages of GC. **f** NR2F1 and NR2F1-AS1 expression level in GC tissues with/without perineural invasion. **g** NR2F1 and NR2F1-AS1 expression level in GC tissues with different M-stages. **h**, **i** Overexpression of NR2F1 and NR2F1-AS1 predicted poor prognosis in GSE62254 cohort. ****P < 0.0001; ***P < 0.001; **P < 0.01; *P < 0.05.

NR2F1-AS1 is an antisense lncRNA (head-to-head) of NR2F1, also known as COUP Transcription Factor I (COUPTF1, Fig. 1a). To explore the biological function of NR2F1 and NR2F1-AS1 in GC, we first analyzed the correlation between gene expression and the clinical characteristics and prognosis of GC. Two independent large cohorts of GC were included in our study, one is the GSE62254 cohort (*n* = 300) and the other is the TCGA cohort (*n* = 373).

**Fig. 2:**
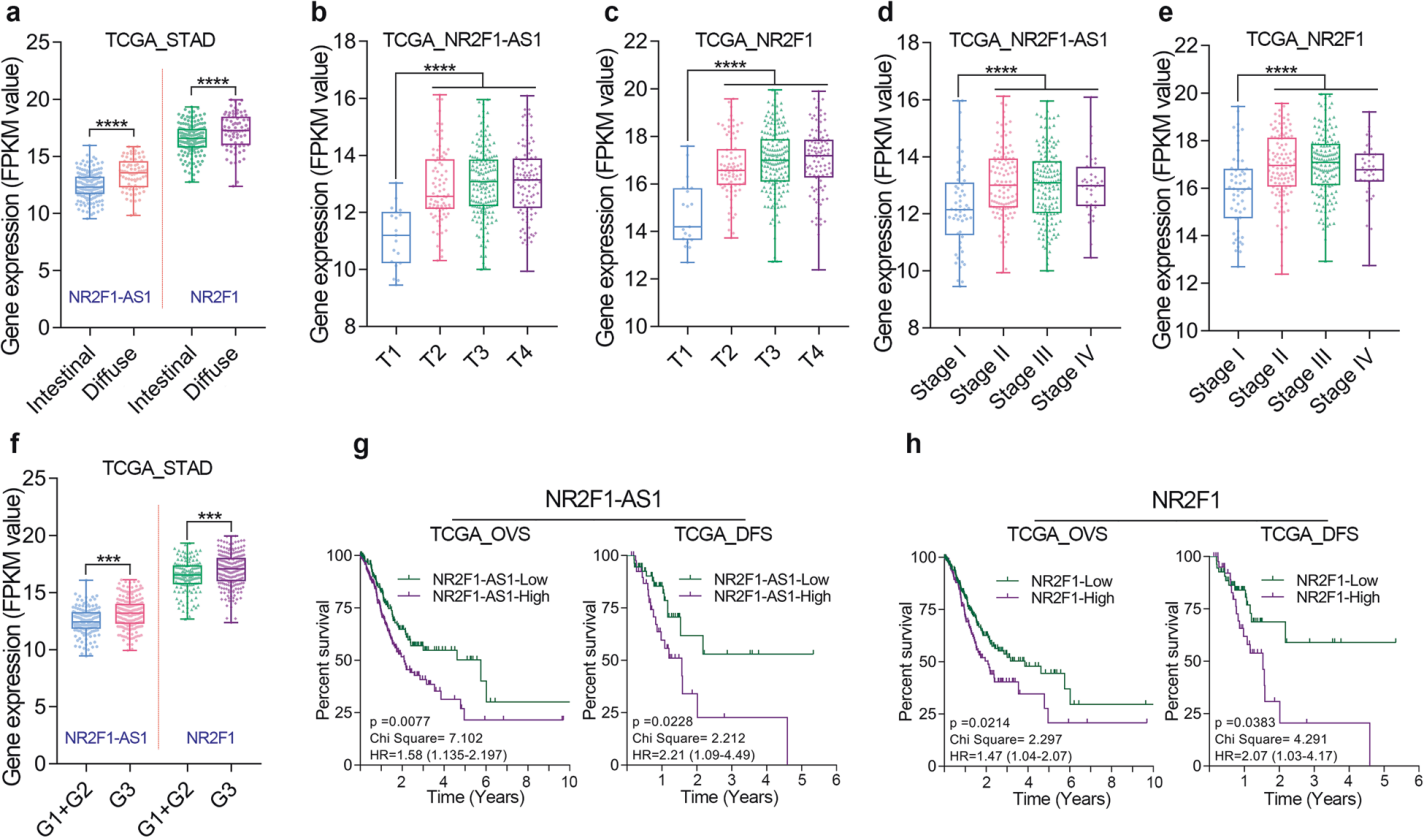
The clinical significance of NR2F1-AS1 and NR2F1 was analyzed in the TCGA cohort. **a** Difference in expression levels of NR2F1-AS1 and NR2F1 between intestinal and diffuse GC tissues. **b**, **c** NR2F1-AS1 and NR2F1 expression level in different T-stages of GC. **d**, **e** NR2F1-AS1 and NR2F1 expression level in different Pathologic stages of GC. **f** Difference in expression levels of NR2F1-AS1 and NR2F1 in GC tissues with different degrees of differentiation. **g**, **h** Overexpression of NR2F1 and NR2F1-AS1 predicted poor prognosis in TCGA cohort. ****P < 0.0001; ***P < 0.001; **P < 0.01; *P < 0.05.

After clinical analysis in GSE62254 cohort, we found that NR2F1 and NR2F1-AS1 were highly expressed in diffuse GC (Fig. 1b). GC patients with relatively high degree of malignancy had higher expression of NR2F1 and NR2F1-AS1 (Fig. 1c–e). Besides, GC tissues with Perineural Invasion (PNI) or distant metastasis tended to possess relatively high expression of NR2F1 and NR2F1-AS1 (Fig. 1f, g). Moreover, GC patients with higher expression of NR2F1-AS1 and NR2F1 had a shorter OV time and DFS time (Fig. 1h, i). Similarly, we also noted that the expression level of NR2F1 and NR2F1-AS1 showed a significant correlation with the histopathological type, malignant progression, poor differentiation, and poor prognosis of GC patients from TCGA cohort (Fig. 2a–h). Based on the clinical analysis in two independent GC cohort, NR2F1-AS1 and NR2F1 are potential promising biomarkers that closely associated with malignant progression and prognosis of GC patients. NR2F1 and NR2F1-AS1 might function oncogenic roles in GC.

### NR2F1-AS1 and NR2F1 are EMT-induced genes in GC

**Fig. 3:**
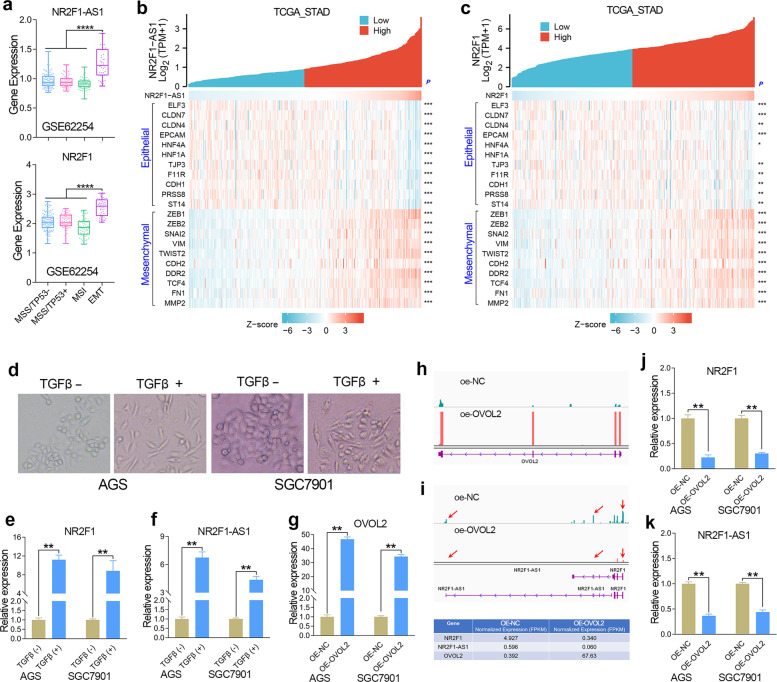
NR2F1-AS1 and NR2F1 was positively regulated by EMT signaling in GC. **a** Expression level of NR2F1-AS1 and NR2F1 in the four subtypes (MSS/TP53−, MSS/TP53+, MSI and EMT) of GC in GSE62254 cohort. **b**, **c** Gene expression correlation analysis confirmed that NR2F1-AS1 and NR2F1 were related to EMT signaling in GC. **d** The changes in the GC cell morphology after TGF-beta treatment. **e**, **f** The expression of NR2F1 and NR2F1-AS1 were determined by qRT-PCR after TGF-beta treatment. **g** The stable overexpression of OVOL2 was verified in GC cell lines. **h** RNA-seq analysis was conducted in GC cells overexpressing OVOL2. **i**–**k** RNA-seq and qRT-PCR analysis showed that the expression of NR2F1 and NR2F1-AS1 were greatly decreased after overexpression of OVOL2. ****P < 0.0001; ***P < 0.001; **P < 0.01; *P < 0.05.

According to the differences of molecular subtypes, GC could be further divided into four subtypes, including MSS/TP53−, MSS/TP53+, MSI, and MSS/EMT subtypes [15]. Our previous publication has identified MAGI2-AS3 as an EMT-related lncRNA in GC [1]. Herein, we confirmed that both NR2F1 and NR2F1-AS1 are EMT-related genes using the same method, since NR2F1 and NR2F1-AS1 were highly expressed in the EMT subtype of GC tissues (Fig. 3a). In addition, correlation analysis based on the gene expression of TCGA cohort showed that the expression of NR2F1 and NR2F1-AS1 were positively correlated with the expression of mesenchymal biomarker genes, but negatively correlated with the expression of epithelial genes in GC (Fig. 3b, c). These results together suggested that both NR2F1 and NR2F1-AS1 were related to EMT signaling in GC.

To further determine whether NR2F1 and NR2F1-AS1 could respond to the EMT signaling, we observed the expression alteration of NR2F1-AS1 and NR2F1 in GC cells with epithelial state or mesenchymal state. TGF-beta is known to be a critical extracellular signal that initiates EMT. Herein, we constructed mesenchymal GC cells by treatment with exogenous TGF-beta (Fig. 3d). After detecting the gene expression by qRT-PCR, we found that both NR2F1 and NR2F1-AS1 were significantly increased after TGF-beta treatment (Fig. 3e, f).

Transcription factor OVOL2 functions as an inducer of MET [16]. Thus, we constructed epithelial-phenotype GC cells by overexpression of OVOL2. According to the qRT-PCR and RNA-seq analysis, the GC cell lines with stable overexpression of OVOL2 were successfully constructed by lentiviral method (Fig. 3g, h). After comparison of gene expression level in GC cell line with/without overexpression of OVOL2 by RNA-seq and qRT-PCR assay, the expression of NR2F1-AS1 and NR2F1 were both found to be sharply declined in the GC cells overexpression of OVOL2 (Fig. 3i–k), indicating that both NR2F1-AS1 and NR2F1 were EMT induced genes.

### NR2F1-AS1 positively regulates the expression of NR2F1 in GC

**Fig. 4:**
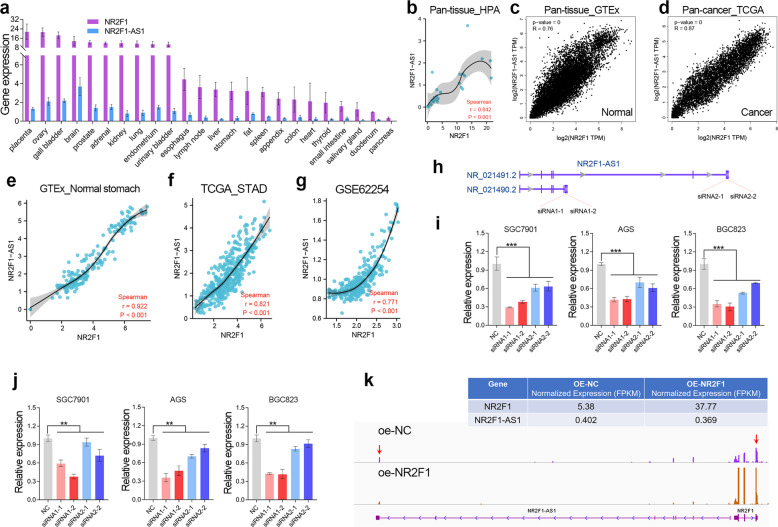
Depletion of NR2F1-AS remarkably compromised the cellular levels of NR2F1. **a** The expression of NR2F1 and NR2F1-AS1 in different human tissues obtained from NCBI website. **b**–**d** High co-expression of NR2F1 and NR2F1-AS1 was observed in pan-tissue and pan-cancer. **e**–**g** NR2F1-AS1 was highly co-expressed with NR2F1 in normal stomach and stomach cancer tissues. **h** The schematic diagram of the siRNAs targeting NR2F1-AS1. **i** The efficiency of knockdown of NR2F1-AS1 was determined by qRT-PCR assay. **j** The expression of NR2F1 was determined after knockdown of NR2F1-AS1 by qRT-PCR assay. **k** RNA-seq analysis showed that NR2F1 overexpression has no obvious effect on the expression of NR2F1-AS1. **P < 0.01.

The quantitative transcriptomics of the Human Protein Atlas (HPA) project provides the tissue specificity of all protein-coding genes and most lncRNAs. According to HPA dataset, NR2F1-AS1 and NR2F1 showed similar tissue-specific expression patterns (Fig. 4a). Pan-tissue and Pan-cancer analysis based on the RNA-seq data in HPA, GTEx and TCGA datasets together showed that NR2F1-AS1 was highly co-expressed with NR2F1 in human tissues (Fig. 4b–d). In normal stomach tissues and GC tissues, NR2F1 and NR2F1-AS1 were also highly co-expressed as expected (Fig. 4e–g).

In order to understand the co-expression between NR2F1 and NR2F1-AS1, we knocked down the expression of NR2F1-AS1 and overexpressed NR2F1 in GC cell lines, respectively. The NR2F1-AS1 has multiple transcript types due to alternative splicing. According to the difference of their last exon, NR2F1-AS1 transcripts could be divided into two groups. Herein, we designed 2 specific siRNAs for each group of NR2F1-AS1 transcript to avoid off-target effect (Fig. 4h). The qRT-PCR assay verified that NR2F1-AS1 was successfully knocked down in GC cell lines (Fig. 4i). Then, we observed a significant decline in the expression of NR2F1 after knockdown of NR2F1-AS1 in GC cell lines (Fig. 4j).

Given that NR2F1 acts as a transcription factor, we also speculated NR2F1-AS1 may be able to be regulated by NR2F1. Hence, we constructed stably overexpressed of NR2F1 by lentiviral method (Fig. 4k). However, according to the RNA-seq analysis, we didn’t observe significant changes in the expression of NR2F1-AS1 in the GC cells overexpressing NR2F1, suggesting NR2F1 cannot regulate NR2F1-AS1 expression in GC. Taken together, the co-expression between NR2F1 and NR2F1-AS1 might be partly due to the positive regulation of NR2F1 by NR2F1-AS1.

### NR2F1-AS1 promotes proliferation and invasion of GC cells in vitro

**Fig. 5:**
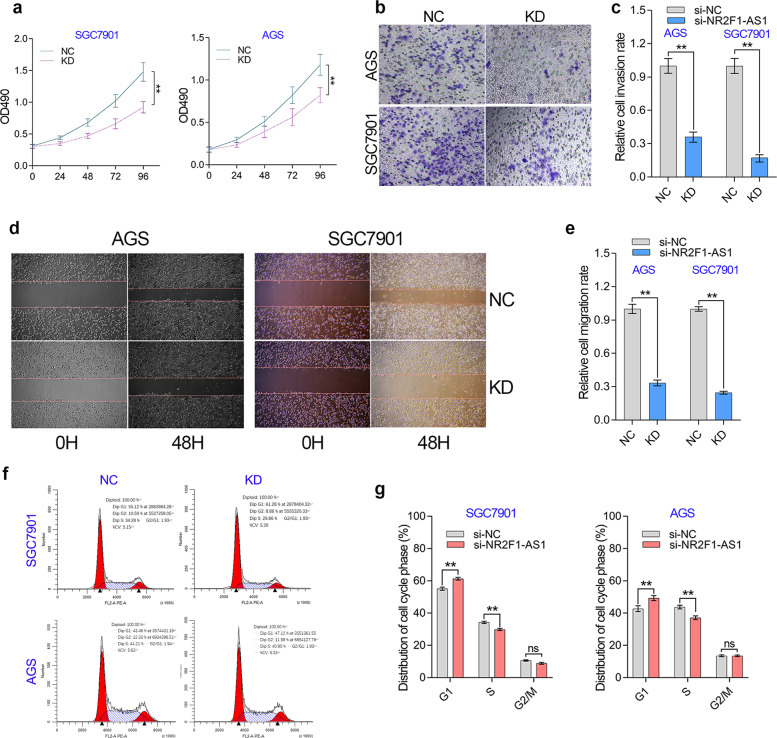
NR2F1-AS knockdown affected cell proliferation, invasion and G1/S transition in GC cell lines. **a** The proliferation of GC cells was determined by MTT assay. **b**, **c** The transwell invasion of GC cells was examined after knockdown of NR2F1-AS1 in GC cell lines. **d**, **e** The migration of GC cells was examined by wound healing assay after knockdown of NR2F1-AS1 in GC cell lines. **f** The effect of NR2F1-AS1 on cell cycle progression was investigated by flow cytometry. NR2F1-AS1 positively regulated the G1/S transition of the cell cycle in GC. **P < 0.0001.

Clinical analysis implied NR2F1-AS1 functioned as an oncogene in GC. Thus, we used loss-of-function study to further verify the effect of NR2F1-AS1 on the biological behavior of GC cells. According to the NR2F1-AS1 knockdown efficiency, we selected two optimal siRNAs for these in vitro experiments. Cell proliferation assay showed that NR2F1-AS1 knockdown suppressed the cell growth of GC cell lines (Fig. 5a). Transwell assay indicated that NR2F1-AS1 knockdown significantly inhibited the cell invasion of GC cell lines (Fig. 5b, c). The wounding healing assay showed that knockdown of NR2F1-AS1 hindered the cell migration of GC cell lines (Fig. 5d, e). Given NR2F1-AS1 knockdown had a significant effect on cell growth, we further investigated the role of NR2F1-AS1 in cell cycle progression of GC cells (Fig. 5f). Knockdown of NR2F1-AS1 caused an obvious G1 arrest in GC cells due to the prolonged G1/S transition, indicating that NR2F1-AS1 may promote GC proliferation through accelerating G1/S transition of GC cells. However, although knockdown of NR2F1-AS1 unexpectedly repressed the early apoptosis and the late apoptosis of GC cells (Fig. S1), it should be pointed out that knockdown of NR2F1-AS1 only inhibited the apoptosis of about 7–9% of GC cells, suggesting that NR2F1-AS1 regulates GC cell apoptosis locally but not extensively.

### NR2F1-AS1 promotes GC progression through maintaining VAMP7 overexpression

**Fig. 6:**
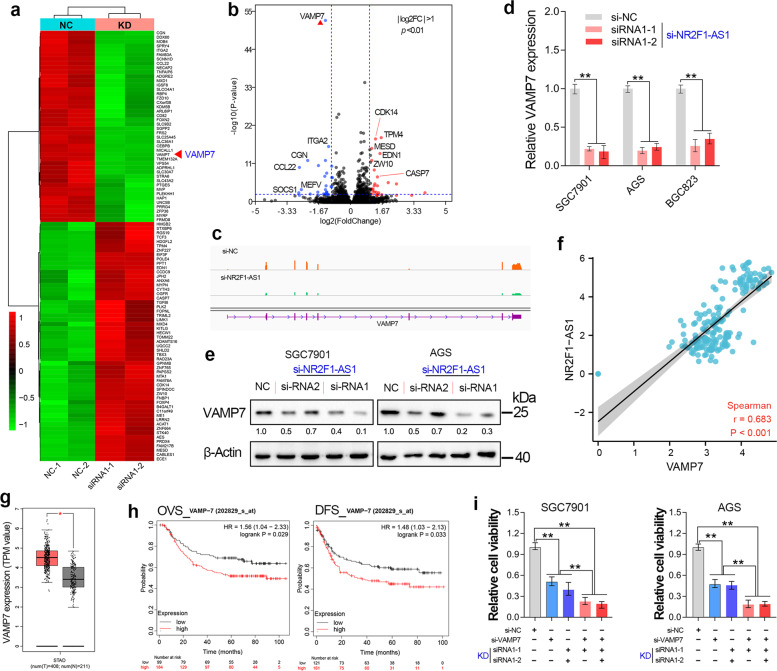
NR2F1-AS1 promotes GC progression through maintaining VAMP7 overexpression. **a** The heat map reveals the top 100 differentially expressed genes after NR2F1-AS1 knockdown. **b** The differentially expressed genes (|Log2FC| > 1, *P* < 0.05) after NR2F1-AS1 depletion were shown in the volcano plot. **c** The transcripts abundance of VAMP7 after NR2F1-AS1 knockdown. **d**, **e** The mRNA level and protein level of VAMP7 were determined after knockdown of NR2F1-AS1. **f** High co-expression between NR2F1-AS1 and VAMP7 was observed in stomach tissues. **g** GEPIA analysis revealed that VAMP7 was upregulated in stomach cancer tissues. **h** Survival analysis indicated that GC patients with relatively high expression of VAMP7 had shorter overall and disease-free survival time. **i** Rescue assay confirmed that NR2F1-AS1 knockdown inhibited GC progression through regulating VAMP7 expression. **P < 0.01.

To explore the molecular mechanism of the cancer-promoting effect of NR2F1-AS1, transcriptome sequencing (GSE183538) was performed in the NR2F1-AS1 depleted GC cells. Two different siRNAs were chosen to avoid target-off effect. After RNA-seq analysis, the top 100 genes that profoundly affect by NR2F1-AS1 knockdown was shown in the heat plot (|Log2FC| > 1, Fig. 6a). The volcano plot shows that knockdown of NR2F1-AS1 had the most significant effect on the expression of VAMP7 gene (Fig. 6b). Besides, the RNA-seq, qRT-PCR and immunoblotting assay together showed that the mRNA level and protein level of VAMP7 were significantly decreased after knockdown of NR2F1-AS1 in GC cell lines (Fig. 6c–e). In addition, NR2F1-AS1 showed a significant co-expression with VAMP7 in normal stomach tissues (Fig. 6f). These results together suggested that NR2F1-AS1 played critical roles in regulating VAMP7 expression in GC.

The biological function of VAMP7 in GC remains largely unknown to date. To verify whether NR2F1-AS1 promotes GC progression by regulating the expression of VAMP7, we first investigated the expression of VAMP7 in normal and stomach cancer tissues. The results showed that VAMP7 was overexpressed in GC (Fig. 6g). To understand the clinical significance of VAMP7 overexpression, the survival curves were analyzed based on the GSE62254 cohort. The results showed that overexpression of VAMP7 predicted poor prognosis in GC (Fig. 6h). These results implied VAMP7 functions as an oncogene in GC. In other words, NR2F1-AS1 might promote GC progression through maintaining VAMP7 overexpression. To verify this possibility, we conducted rescue experiments in GC cell lines. The rescue experiments showed that knockdown of VAMP7 alone significantly inhibited the growth of GC cells, and knockdown of VAMP7 and NR2F1-AS1 deepened the inhibition of cell growth by NR2F1-AS1 knockdown alone (Fig. 6i).

### NR2F1-AS1 functions as a ceRNA to regulate VAMP7 expression by sponging miR-29a

**Fig. 7:**
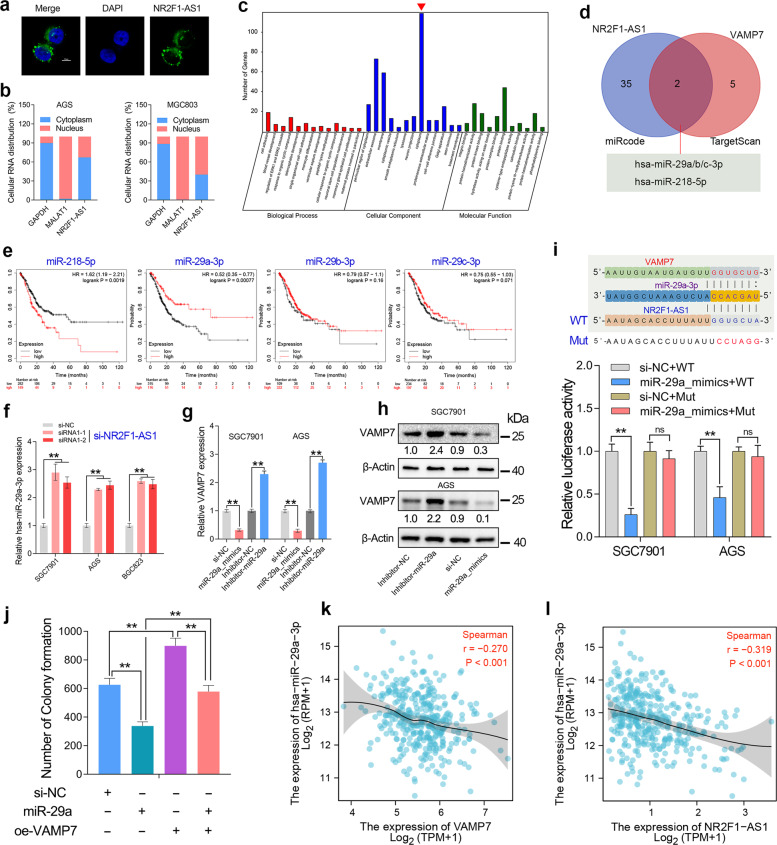
NR2F1-AS1 acted as a sponge of miR-29a-3p to regulate VAMP7 expression. **a** The subcellular distribution of NR2F1-AS1 in SGC7901 cells. **b** NR2F1-AS1 transcripts were located in cytoplasm and nucleus of GC cells. **c** GO analysis confirmed NR2F1-AS1 knockdown mainly function in cytoplasm. **d** The miRNAs that sponged by NR2F1-AS1 and miRNAs that targeting VAMP7 were predicted by miRCode and TargetScan. **e** The survival analysis of miR-218-5p and miR-29a/b/c-3p in GC. **f** Knockdown of NR2F1-AS1 significantly upregulated miR-29a-3p expression in GC cell lines. **g**, **h** VAMP7 was negatively regulated by miR-29a-3p in GC cell lines. **i** Luciferase reporter assay confirmed that miR-29a-3p can bind to NR2F1-AS1. **j** Rescue assay confirmed that miR-29a-3p inhibited the colony formation of GC cells by targeting VAMP7. **k**, **l** Both NR2F1-AS1 and VAMP7 showed a significant negative expression correlation with miR-29a-3p in the GC cohort from TCGA. **P < 0.01.

The biological function of lncRNA is closely related to its subcellular location [17]. Therefore, RNA Fish assays was conducted in GC cells. The results revealed that NR2F1-AS1 transcripts were distributed in the nucleus and cytoplasm of SGC7901 cells (Fig. 7a). Besides, the nuclear-cytoplasmic RNA fractionation assay followed by qRT-PCR showed that more than half of NR2F1-AS1 transcripts were located in the cytoplasm of GC cell lines (Fig. 7b). GO analysis showed that NR2F1-AS1 mainly functions in cytoplasm (Fig. 7c).

Emerging studies have reported the biological function of NR2F1-AS1 in cancers. Most of the studies showed that NR2F1-AS1 promoted tumor progression by function as a ceRNA in breast cancer [18], thyroid cancer [19], and lung cancer [20]. Our subcellular localization and GO analysis also suggested that NR2F1-AS1 had the basic conditions for exerting ceRNA roles. Therefore, we analyzed the miRNAs that might be sponged by NR2F1-AS1 and the miRNAs that predicted to target VAMP7. After taking the intersection, we found that NR2F1-AS1 may regulate VAMP7 expression by sponging miR-29a/b/c and miR-218 (Fig. 7d). However, survival analysis showed that miR-218 predicted poor prognosis in GC, while only miR-29a predicted favorable prognosis in GC (Fig. 7e). Besides, increasing studies have reported the tumor-suppressive role of miR-29a-3p in GC. Knockdown of NR2F1-AS1 significantly increased the expression level of miR-29a-3p in GC cell lines (Fig. 7f). Taken together, we considered NR2F1-AS1 might regulate VAMP7 expression by sponging miR-29a-3p in GC. Therefore, we investigated the effect of miR-29a-3p on the expression level of VAMP7 in GC. The results showed that VAMP7 was downregulated in the GC cells transfecting with miR-29a mimics, but was upregulated in the GC cells transfecting with miR-29a inhibitors (Fig. 7g, h). Moreover, dual-luciferase reporter assay and rescue assay together confirmed that miR-29a-3p repressed cell proliferation by targeting VAMP7 in GC (Fig. 7i, j). Additionally, both NR2F1-AS1 and VAMP7 showed a significant negative correlation with miR-29a in the TCGA cohort (Fig. 7k, l), suggesting there is a novel ceRNA network composed of NR2F1-AS1, miR-29a-3p and VAMP7 in GC.

**Fig. 8:**
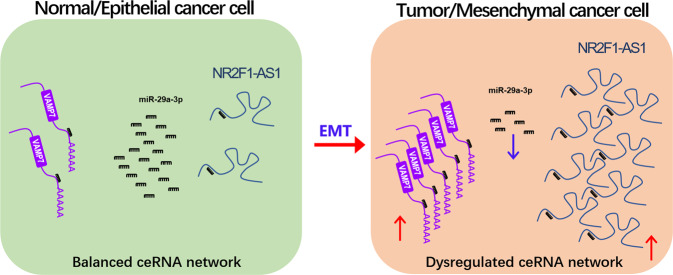
Hypothetical working model of lncRNA NR2F1-AS1 in GC. NR2F1-AS1 expression was greatly upregulated in GC, especially in metastatic GC cells with mesenchymal phenotypes. In the normal gastric cells or the epithelial GC cells, the ceRNA network, composed of NR2F1-AS1, miR-29a-3p and VAMP7, is balanced. After tumorigenesis or initiation of EMT, the ceRNA network is dyregulated, resulting in overexpression of the oncogene VAMP7, which ultimately drives GC progression.

In summary, lncRNA NR2F1-AS1 acts as a sponge for miR-29a-3p and promotes GC progression through regulating miR-29a-3p/VAMP7 axis. Additionally, NR2F1-AS1 was positively regulated by EMT signaling. Once abnormal EMT signaling occurs, it will lead to a significant up-regulation of NR2F1-AS1, leading to the deregulated ceRNA network and aberrant VAMP7 expression, and ultimately promoting the malignant progression of GC (Fig. 8).

## Discussion

Large number of non-coding RNAs (ncRNA) and protein-coding genes have been found to be dysregulated in tumors or other diseases. In the past decades, the mechanism of dysregulated ncRNAs has received little attention. Recently, increasing studies have reported ncRNAs can regulate tumor progression through forming ceRNA networks [21]. The components in ceRNA network are usually interrelated. Aberrant expression of any network component may derail complex regulatory circuits and ultimately result in tumorigenesis and metastasis [22]. Therefore, identification of ceRNA network is essential to understand how cancer-related genes are dysregulated, thereby improving disease prognosis. Since ceRNA network can at least partially explain how cancer-related genes are dysregulated, exploring and identifying novel ceRNA networks is essential to improve the prognosis of the disease.

In the present study, we identified a novel ceRNA network composed of NR2F1-AS1, miR-29a-3p, and VAMP7 in GC by transcriptome sequencing. Clinical analysis revealed that NR2F1-AS1 showed a significant correlation with the degree of malignancy and predicted poor prognosis in two independent GC cohorts. Loss-of-function studies indicated that NR2F1-AS1 promotes the proliferation, migration and invasion of GC cells. Similarly, VAMP7, a novel target gene of miR-29a-3p, was overexpressed and predicted poor prognosis in GC (Fig. 6g–l). Rescue assay has further confirmed that NR2F1-AS1 promotes GC progression through miR-29a/VAMP7 axis. In other words, during the current ceRNA network, NR2F1-AS1 and VAMP7 played oncogenic roles in GC, while increasing studies have reported that miR-29a-3p exerted tumor-suppressive roles in GC [23–26]. Taken together, this deregulated ceRNA network plays critical roles in regulating GC progression.

Divergent lncRNAs usually co-expressed with nearby protein-coding genes [27]. On the one hand, the antisense lncRNA shares a promoter with neighboring genes, which causes them to be regulated by the same transcription factors [28]. On the other hand, emerging evidences reported that the antisense lncRNA and neighboring genes can be stabilized together by forming RNA–RNA duplex with the overlapping sequences of their transcripts [21, 29]. In addition, accumulating evidences showed that antisense lncRNAs can participate the transcription of nearby genes by recruiting transcription-related proteins or affecting R-loop formation, or regulating the methylation of enhancers [30–33]. Herein, we also observed a high co-expression between NR2F1-AS1 and NR2F1 in pan-cancer and pan-tissue. Given that NR2F1-AS and NR2F1 were together induced by EMT signaling (Fig. 3) and depletion of NR2F1-AS1 inhibited NR2F1 expression (Fig. 4), we considered that the co-expression between NR2F1-AS1 and NR2F1 was because they share the promoter and the transcriptional regulation of NR2F1-AS1 on NR2F1. Interestingly, a recent study has reported that NR2F1-AS1 expression was induced by NR2F1 in esophageal squamous cell carcinoma [34]. However, according to our qRT-PCR and RNA-seq analysis, NR2F1 overexpression has no obvious effect on the expression of NR2F1-AS1 in GC (Fig. 4k).

The relationship between EMT and NR2F1/NR2F1-AS1 has not been reported yet. Our previous work has identified a EMT-related lncRNA MAGI2-AS3 in GC [1]. In this study, we further identified both NR2F1 and NR2F1-AS1 as EMT-induced genes in GC, since NR2F-AS1 and NR2F1 were both positively regulated by EMT signaling. Consistent with our findings, Feng et al. also reported that NR2F1 is greatly declined in GC cells that silenced Twist1, a well-known EMT-related transcription factor (EMT-TF) [35]. These findings together suggested NR2F1 was positively regulated by EMT signaling. However, after analysis of RNA-seq data of NR2F1-AS1 knockdown, we found NR2F1-AS1 knockdown has no significant effect on the expression of EMT-related biomarker genes, such as E-cadherin, Vimentin, and ZEB1. However, the specific role of NR2F1 during the EMT process remains to be further explored in GC.

## Conclusions

LncRNA NR2F1-AS1 and NR2F1 predicted poor prognosis in GC. Both NR2F1-AS1 and NR2F1 are identified to be EMT-induced genes in GC. The co-expression of NR2F1 and NR2F1-AS1 may be partly due to the regulation of NR2F1 by NR2F1-AS1. A novel ceRNA network composed of NR2F1-AS1, miR-29a-3p, and VAMP7 was identified in GC. Our finding highlights that oncogenic lncRNA NR2F1-AS1 promotes GC metastasis through regulating miR-29a/VAMP7 signal axis.

## Materials and methods

### Microarray data analysis of GSE62254 and pan-cancer analysis in TCGA

The gene expression data in GSE62254 used in this study was downloaded from the NCBI web server. The clinical information of GC patients in GSE62254 cohort was download as we described previously [12]. RNA-Seq data of 407 gastric cancer samples and the correlated clinical information of 443 gastric cancer samples were downloaded from the Cancer Genome Atlas (TCGA). Expression level of each gene was calculated from log2 of its upper quartile FPKM (FPKM-UQ) value.

### Cell transfection and establishment of cell lines

Human gastric cancer cell lines were purchased from GeneChem (Shanghai, China). The siRNAs listed in Table S1 were designed and synthesized by Genepharma (Shanghai, China). The lentiviruses for overexpression of NR2F1 and OVOL2 in GC cell lines were purchased from GeneChem (Shanghai, China). For TGF-beta treatment, 10 ng/ml recombinant TGF-β1 (cat. no. HZ-1011; Proteintech, Wuhan, China) was added to the medium. GC cells were cultured for at 37 °C as usual. At the indicated time points, the cells were harvested for mRNA and protein analysis as well as for other assays.

### RNA sequencing

The total RNA in GC cells was extracted to perform RNA sequencing (RNA-seq). A total amount of 1.5 µg RNA per sample was used as input material for the RNA sample preparations. The whole step of library construction and sequencing was performed at Shanghai Lifegenes Technology Co., Ltd. The RNA-seq data was uploaded in Table S2. The GEO accession number is GSE183538.

### Subcellular location of lncRNA

The Nuclear/cytoplasmic RNA isolation and RNA FISH assay was conducted as we previously described [21]. For Nuclear/cytoplasmic RNA isolation, cytoplasm RNA and nuclear RNA were extracted using the nuclear-cytoplasmic separation kit (BB-36021-2, BestBio, China). After Quantitative RT-PCR, comparative Δ*C*_t_ method was used to examine the relative distribution of RNA. For RNA FISH assay, The 5′FAM-NR2F1-AS1 probes were designed and synthesized by Sangon Biotech (Shanghai). After incubation and hybridization, images were taken with a confocal microscope (Zeiss).

### RNA isolation and quantitative RT-PCR

Total RNA was extracted using Trizol reagent (Invitrogen, USA). Reverse transcription was performed to obtain cDNA by using the PrimeScript^TM^ RT reagent Kit (Perfect Real Time, Takara). The qPCR protocol was using One Step TB Green PrimeScript^TM^ RT-PCR Kit II (Takara) according to the manufacturer’s instructions. The qPCR analysis was conducted on Bio-Rad CFX Manager 3.1 real-time PCR system. All the primers listed in Table S1 were synthesized by Wcgene Biotech (Shanghai, China). RNU6B (U6) and ACTB were used as internal controls. Each gene was run in triplicate. Relative fold changes of gene expression were calculated using the comparative ^ΔΔ^Ct method. All primers listed in Table S1 were synthesized by Wcgene Biotech (Shanghai, China).

### Western blot assay

Gastric cancer cells were lysed in RIPA buffer added 1 mM PMSF. Approximately 100 μg of total protein was electrophoresed through 10% SDS polyacrylamide gels and were then transferred to a PVDF membrane. After blocking with 5% skimmed milk at 4 °C for 1 h, the membrane was incubated with primary antibody at 4 °C overnights. The blots were then washed and incubated with horseradish peroxidase (HRP)-conjugated secondary antibody (1:10,000, Earthox) for 1.5 h at room temperature. Detection was performed by using a SuperLumia ECL HRP Substrate Kit (Abbkine) and visualized using a Bio-Rad Imaging System (USA). The VAMP7 antibody used in this study was purchased from ABclonal (A18698, Wuhan, China).

### Dual luciferase reporter assay

The wildtype and mutant NR2F1-AS1 fragment were amplified by PCR and ligated into the pEZX-FR01-dual luciferase reporter vector (GeneCopoeia, USA). GC cells were seeded into 12-well-tissue plates 24 h before transfection, and then co-transfected with 5 ng siRNA and 1 mg plasmid using the Lipofectamine 2000 Reagent (Invitrogen), according to the manufacturer’s instructions. After another 48 h, cells were assayed using the Dual-Luciferase reporter assay system kit (GeneCopoeia, USA). All experiments were performed in triplicate and data were pooled from three independent experiments.

### Flow cytometry assay

After 48 h transfected with siRNAs and corresponding negative control siRNAs, SGC7901, and AGS cells were collected and performed cell cycle assay and cell apoptosis assay in accordance with the manufacture’s protocol (BB-4104, BestBio, China). Flow cytometry assays were performed on the CytoFLEX machine (Beckman, USA). The cell cycle and cell apoptosis distribution were quantified using the CytExpert software.

### Statistical analysis

For gene expression analysis of different subtypes of GC, the P values were estimated using Mann–Whitney nonparametric test. Survival curves were calculated using the Kaplan–Meier method, and differences between the curves were analyzed using the log-rank test. All the rest of the experiments were used unpaired *t*-test or one-way ANOVA test. For all experiments, a minimum of triplicates per group and repetition of at least three times was applied to achieve reproducibility. All the gene expression correlation analysis were used spearman test. The regression line was generated by corresponding R package. All tests with *P* values less than 0.05 considered statistically significant.

## Supplementary information

Changes to the author list (informed consent)

Supplementary legends

Reproducibility checklist

Figure S1

Table S1

Table S2

## Data Availability

The datasets generated during the current study are available in the GEO repository (GSE183538).
