# Supplementary material for: The EMT-induced lncRNA NR2F1-AS1 positively modulates NR2F1 expression and drives gastric cancer via miR-29a-3p/VAMP7 axis
Source: Cell Death Dis. 2022 Jan 26;13(1):84. doi: 10.1038/s41419-022-04540-2 (PMC8791943; doi:10.1038/s41419-022-04540-2)
Supplement: Supplementary file 1 — Changes to the author list (informed consent) [file 41419_2022_4540_MOESM1_ESM.pdf]

Changes to the author list

Manuscript No. CDDIS-21-3737

Article Entitled: The EMT-induced lncRNA NR2F1-AS1 positively modulates NR2F1 expression and drives gastric cancer via miR-29a-3p/VAMP7 axis

Author information:

Dandan Li [1,2], Mengjie Xu [3], Zidi Wang [1,2], Pan Huang [1,2], Congcong Huang [1,2], Zhen Chen [2], Gaijuan Tang [4], Xingji Zhu [2], Mengyu Cai [2] and Shanshan Qin [1,2]*

[1] Hubei Key Laboratory of Embryonic Stem Cell Research, School of Basic Medical Sciences, Hubei University of Medicine, Shiyan 442000, Hubei, P.R. China

[2] Laboratory of Tumor biology, Academy of Bio-Medicine Research, Hubei University of Medicine, Shiyan 442000, Hubei, P.R. China

[3] Department of Endocrinology, Taihe Hospital, Hubei University of Medicine, Shiyan 442000, Hubei, P.R. China

[4] College of Plant Protection, Northwest A&F University, Yangling 712100, China

* **Corresponding author:**

Shanshan Qin, Email: qinss77@163.com

Dear authors:

Because of the contributions by WZD, TGJ and ZXJ in the revision stage, we need to change the current manuscript's author order and addresses, as listed above.

Among the authors in the list, QSS designed experiments, offered direction and help on the whole project. LDD, XMJ, WZD, HP, HCC, CMY, ZXJ and CZ conducted the experiments, analyzed the results. QSS, LDD and TGJ performed bioinformatics analysis. QSS and LDD drafted the manuscript. QSS and LDD reviewed the manuscript and made significant revisions on the drafts.

After consultations, all the authors agreed with the addition of authors in this paper, and all the authors agreed with the rearrangement of the names.

If everyone have no comments on the change of authorship of this manuscript, please email to me with "I agree to these changes".

Thank you very much for your attention, looking forward to your reply

Best Wishes

Correspondence to Shanshan Qin, Ph. D.

Hubei Key Laboratory of Embryonic Stem Cell Research

Hubei University of Medicine

Renmin Road 30, Maojian District

Shiyan, Hubei 442000, P.R. China

The original email records:

发件人： qinss77<qinss77@163.com> +

收件人： lidandan_cup<lidandan_cup@163.com> taihexumengjie<taihexumengjie@163.com> hbmuwzd<hbmuwzd@163.com> panhuang<panhuang@hbmu.edu.cn>
chenzhen000608<chenzhen000608@163.com> tangrui-happy<tangrui-happy@163.com> 3340308693<3340308693@qq.com> 505960726<505960726@qq.com

时　间： 2022年01月12日 11:21 (星期三)

发送状态： 发送成功 查看详情

100GB阿里云盘免费试用，不限速就是快！ 注册领取

Changes to the author list

Manuscript No. CDDIS-21-3737

Article Entitled: The EMT-induced lncRNA NR2F1-AS1 positively modulates NR2F1 expression and drives gastric cancer via miR-29a-3p/VAMP7 axis

Author information:

Dandan Li [1,2], Mengjie Xu [3], Zidi Wang [1,2], Pan Huang [1,2], Congcong Huang [1,2], Zhen Chen [2], Gaijuan Tang [4], Xingji Zhu [2], Mengyu Cai [2] and Shanshan Qin [1,2*]

[1] Hubei Key Laboratory of Embryonic Stem Cell Research, School of Basic Medical Sciences, Hubei University of Medicine, Shiyan 442000, Hubei, P.R. China

Author information:

Dandan Li [1,2], Mengjie Xu [3], Zidi Wang [1,2], Pan Huang [1,2], Congcong Huang [1,2], Zhen Chen [2], Gaijuan Tang [4], Xingji Zhu [2], Mengyu Cai [2] and Shanshan Qin [1,2*]

[1] Hubei Key Laboratory of Embryonic Stem Cell Research, School of Basic Medical Sciences, Hubei University of Medicine, Shiyan 442000, Hubei, P.R. China

[2] Laboratory of Tumor biology, Academy of Bio-Medicine Research, Hubei University of Medicine, Shiyan 442000, Hubei, P.R. China

[3] Department of Endocrinology, Taihe Hospital, Hubei University of Medicine, Shiyan 442000, Hubei, P.R. China

[4] College of Plant Protection, Northwest A&F University, Yangling 712100, China

* Corresponding author:

Shanshan Qin, Email: qinss77@163.com

Dear authors:

Because of the contributions by WZD, TGJ and ZXJ in the revision stage, we need to change the current manuscript's author order and addresses, as listed above.

Among the authors in the list, QSS designed experiments, offered direction and help on the whole project. LDD, XMJ, WZD, HP, HCC, CMY, ZXJ and CZ conducted the experiments, analyzed the results. QSS, LDD and TGJ performed bioinformatics analysis. QSS and LDD drafted the manuscript. QSS and LDD reviewed the manuscript and made significant revisions on the drafts.

After consultations, all the authors agreed with the addition of authors in this paper, and all the authors agreed with the rearrangement of the names.

If everyone have no comments on the change of authorship of this manuscript, please email to me with "I agree to these changes".

Thank you very much for your attention, looking forward to your reply

[illegible]

Re: Changes to the author list（CDDIS-21-3737)

发件人：李丹丹<lidandan_cup@163.com>

收件人：qinss77<qinss77@163.com> +

时　间：2022年01月12日 23:47 (星期三)

100GB阿里云盘免费试用，不限速就是快！ 注册领取

I agree to these changes.

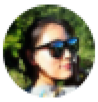

李丹丹

邮箱：lidandan_cup@163.com

签名由 网易邮箱大师 定制

On 01/12/2022 11:21, qinss77 wrote:

Changes to the author list

Re: Changes to the author list（CDDIS-21-3737）

发件人：hbmuwzd<hbmuwzd@163.com>

收件人：qinss77<qinss77@163.com> +

时　间：2022年01月12日 12:40 (星期三)

100GB阿里云盘免费试用，不限速就是快！ 注册领取

I agree to these changes

hbmuwzd

邮箱：hbmuwzd@163.com

签名由 网易邮箱大师 定制

On 01/12/2022 11:21, qinss77 wrote:

Changes to the author list

Re:Re: Changes to the author list（CDDIS-21-3737）

发件人：唐改娟<tangrui-happy@163.com>

收件人：我<qinss77@163.com> +

时　间：2022年01月12日 12:10 (星期三)

100GB阿里云盘免费试用，不限速就是快！ 注册领取

I agree to these changes.

Re: Changes to the author list（CDDIS-21-3737)

发件人： huangcong163<huangcong163@126.com>

收件人： qinss77<qinss77@163.com> +

时　间： 2022年01月12日 11:49 (星期三)

100GB阿里云盘！注册免费试用 注册领取

I agree to these changes.

发自我的iPhone

------------------ Original ------------------

From: qinss77 <qinss77@163.com>

Date: Wed,Jan 12,2022 11:21 AM

To: lidandan_cup <lidandan_cup@163.com>, taihexumengjie <taihex
<chenzhen000608@163.com>, tangrui-happy <tangrui-happy@163.c

Subject: Re: Changes to the author list（CDDIS-21-3737）

Re:Changes_to_the_author_list（CDDIS-21-3737）

发件人：黄盼<20200509@hbmu.edu.cn>

收件人：qinss77<qinss77@163.com>

时　间：2022年01月12日 11:48 (星期三)

100GB阿里云盘免费试用，不限速就是快 注册领取

I agree to these changes.

---------- Origin message ----------

>From："qinss77" <qinss77@163.com>

>To：lidandan_cup@163.com, taihexumengjie@163.com, hbmuwzd@16
505960726@qq.com

>Subject：Changes_to_the_author_list（CDDIS-21-3737）

>Date：2022-01-12 11:21:00

> Changes to the author list

Re: Changes to the author list（CDDIS-21-3737）

发件人：徐梦婕<taihexumengjie@163.com>

收件人：qinss77<qinss77@163.com> +

抄送人：lidandan_cup@163.com<lidandan_cup@163.com> hbmuwzd@163.com

chenzhen000608@163.com<chenzhen000608@163.com> ↓还有3个联系人

时　间：2022年01月12日 11:47 (星期三)

翻译成中文

100GB阿里云盘免费试用，不限速就是快 注册领取

I agree to these changes

taihexumengjie

邮箱：taihexumengjie@163.com

签名由 网易邮箱大师 定制

Re: Changes to the author list（CDDIS-21-3737）

发件人：505960726<505960726@qq.com>
收件人：Shanshan Qin<qinss77@163.com>
时　间：2022年01月12日 11:38 (星期三)

100GB阿里云盘免费试用，不限速就是快！ 注册领取

I agree to these changes

蔡梦雨

邮箱：505960726@qq.com

签名由 网易邮箱大师 定制

On 01/12/2022 11:21, qinss77 wrote:

Changes to the author list

Re: Changes to the author list（CDDIS-21-3737)

发件人：佑你喜乐<3340308693@qq.com>

收件人：qinss77<qinss77@163.com>

时　间：2022年01月12日 11:29 (星期三)

100GB阿里云盘免费试用，不限速就是快 注册领取

I agree to these changes

---Original---

From: "qinss77"<qinss77@163.com>
Date: Wed, Jan 12, 2022 11:21 AM
To: "lidandan_cup"<lidandan_cup@163.com>;"taihexumengjie"<taihexumengjie@163.com>
<chenzhen000608@163.com>;"tangrui-happy"<tangrui-happy@163.com>;"3340308693"<3
Subject: Changes to the author list（CDDIS-21-3737）

Changes to the author list

Manuscript No. CDDIS-21-3737

Re: Changes to the author list（CDDIS-21-3737）

发件人：18卓越医师班学委陈珍<chenzhen000608@163.com>

收件人：qinss77<qinss77@163.com> +

时　间：2022年01月12日 11:25 (星期三)

翻译成中文

100GB阿里云盘免费试用，不限速就是快！ 注册领取

I agree to these changes

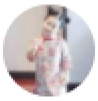

chenzhen000608

邮箱：chenzhen000608@163.com

签名由 网易邮箱大师 定制

On 01/12/2022 11:21, qinss77 wrote:
