# Supplementary material for: The EMT-induced lncRNA NR2F1-AS1 positively modulates NR2F1 expression and drives gastric cancer via miR-29a-3p/VAMP7 axis
Source: Cell Death Dis. 2022 Jan 26;13(1):84. doi: 10.1038/s41419-022-04540-2 (PMC8791943; doi:10.1038/s41419-022-04540-2)
Supplement: Supplementary file 2 — Supplementary legends [file 41419_2022_4540_MOESM2_ESM.docx]

**Supplementary Files**

**Table S1** The primers and siRNAs used in this study.

**Table S2** The normalized RNA-seq data of NR2F1-AS1 knockdown in GC cell lines.

**Figure S1** The percentage of apoptotic cells was determined by flow cytometric analysis (left panel). The statistical results were shown on the right panel.
