# Supplementary figures and images for: The EMT-induced lncRNA NR2F1-AS1 positively modulates NR2F1 expression and drives gastric cancer via miR-29a-3p/VAMP7 axis
Source: Cell Death Dis. 2022 Jan 26;13(1):84. doi: 10.1038/s41419-022-04540-2 (PMC8791943; doi:10.1038/s41419-022-04540-2)

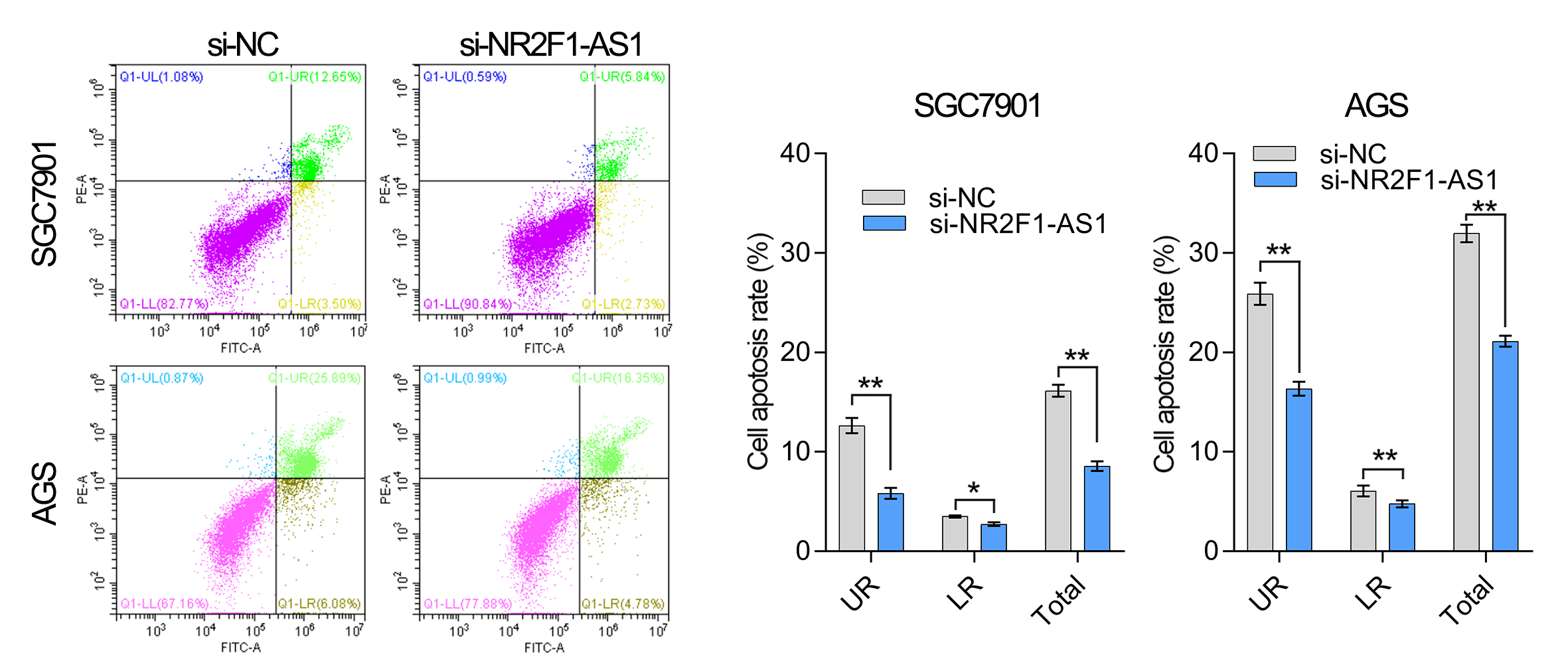

Supplement: Supplementary file 4 — Figure S1 [file 41419_2022_4540_MOESM4_ESM.tif]
