# Supplementary material for: The EMT-induced lncRNA NR2F1-AS1 positively modulates NR2F1 expression and drives gastric cancer via miR-29a-3p/VAMP7 axis
Source: Cell Death Dis. 2022 Jan 26;13(1):84. doi: 10.1038/s41419-022-04540-2 (PMC8791943; doi:10.1038/s41419-022-04540-2)
Supplement: Supplementary file 5 — Table S1 [file 41419_2022_4540_MOESM5_ESM.docx]

**Table S1.** Primers and siRNAs used in this study.

| Gene Name | Sequence (5’→3’) | |
| --- | --- | --- |
| The sequence of siRNAs used in this study | | |
| siRNA name | Sense strand | Antisense strand |
| si-NC (Universal) | CCGAGGAUCUUGGGAUCAUTT | AUGAUCCCAAGAUCCUCGGTT |
| siRNA1#1 | GGUGUUCGAUAUUCUUCUUTT | AAGAAGAAUAUCGAACACCTT |
| siRNA1#2 | CCUGAAGGACAAGUGGUCUTT | AUGAGAGCCUAACUAAUGCTT |
| siRNA2#1 | GCAUUAGUUAGGCUCUCAUTT | CUUUAAGUAGGUUUCUUGCTT |
| siRNA2#2 | GCUCUAAUUGGACUGGUAATT | UUACCAGUCCAAUUAGAGCTT |
| miRNA mimics-NC | UUCUCCGAACGUGUCACGUTT | ACGUGACACGUUCGGAGAATT |
| miR-29a-5p mimics | UAGCACCAUCUGAAAUCGGUUA | ACCGAUUUCAGAUGGUGCUAUU |
| Inhibitors-NC | CAGUACUUUUGUGUAGUACAA | |
| miR-29a-5p inhibitors | UAACCGAUUUCAGAUGGUGCUA | |
| The sequence of RT (reverse transcription) and qPCR (quantification PCR) primers used in this study | | |
|  | Forward primer | Reverse primer |
| NR2F1_qPCR | ATCCGAGCTACAAAGCATGG | TCCACATCCGTCCACAATAA |
| NR2F1-AS1_qPCR | GCCCATGATGAACCTGTTTT | TTACATCACGGCATGGTAGC |
| OVOL2_qPCR | CGCCAGATCGAAAATCAAGT | GTAGGGACGAATGCCTGTGT |
| VAMP7_qPCR | TGTTTTCCCATTGCAGTTGA | ACCTCCCTTTGCACAGCTAA |
| ACTIN_qPCR | ATCGTCCACCGCAAATGCTTCTA | AGCCATGCCAATCTCATCTTGTT |
| miR-29a_RT | GTCGTATCCAGTGCGTGTCGTGGAGTCGGCAATTGCACTGGATACGACTAACCGA | |
| miR-29a_qPCR | GGGGTAGCACCATCTGAAATC | CAGTGCGTGTCGTGGAGT |
| U6_ qPCR | CTCGCTTCGGCAGCACA | AACGCTTCACGAATTTGCGT |
| U6_RT | AACGCTTCACGAATTTGCGT | |
